# 

**Published:** 1919-03

**Authors:** 


					﻿VOL. XXXIV.
MARCH-—
No. 9.
Texas
Medical
Journal
Austin, Texas, as second-class mail matter
				

